# The association of maternal folic acid supplementation and prenatal folate and vitamin B12 concentrations with child dental development

**DOI:** 10.1111/cdoe.12620

**Published:** 2021-01-24

**Authors:** Brunilda Dhamo, Vincent W. V. Jaddoe, Eric A. P. Steegers, Eppo B. Wolvius, Edwin M. Ongkosuwito

**Affiliations:** ^1^ Department of Oral & Maxillofacial Surgery Special Dental Care and Orthodontics Erasmus University Medical Centre Rotterdam The Netherlands; ^2^ The Generation R Study Group Erasmus University Medical Centre Rotterdam The Netherlands; ^3^ Department of Epidemiology Erasmus University Medical Centre Rotterdam The Netherlands; ^4^ Department of Obstetrics & Gynecology Erasmus University Medical Centre Rotterdam The Netherlands

**Keywords:** B vitamins, folate, supplements, tooth maturation, vitamin B12

## Abstract

**Objective:**

Low folic acid, folate and vitamin B12 might affect tooth formation and mineralization. The conversion of folic acid into folate is catalysed by the methylenetetrahydrofolate (MTHFR) enzyme which is encoded by the *MTHFR* gene. Among 3728 mothers and their 10‐year‐old children from the Generation R Study, we investigated associations of maternal folic acid supplementation and prenatal folate and vitamin B12 concentrations with child dental development. Secondly, we checked the modifying effect of *MTHFR‐C677T* polymorphism.

**Methods:**

Information on folic acid supplementation was obtained by questionnaires. Concentrations of folate and vitamin B12 were measured from venous samples taken in early pregnancy. Developmental stages of teeth were defined by the Demirjian method at the age‐10 assessment. In addition, dental age of the children was calculated using the Dutch standard. GLM and multivariate linear regression models were built to study the associations.

**Results:**

Folic acid supplementation started when pregnancy was known (*β* = −0.09; 95% CI: −0.17, −0.01) and folic acid supplementation started prior to known pregnancy (*β* = −0.12; 95% CI: −0.20, −0.04) were both associated with decelerated dental development by 1‐2 months lower dental age of 10‐year‐old children. Folate (*β* = −0.02, 95% CI: −0.05, 0.02) and vitamin B12 (*β* = 0.03, 95% CI: −0.00, 0.06) were not associated with dental age. *MTHFR‐C677T* did not modify the associations.

**Conclusions:**

Maternal folic acid supplementation delays dental development of children by 1‐2 months dental age, whereas maternal folate and vitamin B12 concentrations in early pregnancy do not affect the timing of child dental development.

## 1 INTRODUCTION

Dental development is defined as a progressive and continuous process determined by epithelial‐mesenchymal interactions and controlled by genetic, epigenetic and environmental factors over time.^1^, ^2^, ^3^ As an environmental source, various micronutrients can contribute to the regulation of dental development.^4^, ^5^ For example, vitamin deprivation during pregnancy and early childhood thoroughly affect the mineralization and maturation of teeth, including the emergence of the primary and permanent dentition.^6^, ^7^, ^8^ The deficiency of folate (vitamin B9) and vitamin B12 in children may be associated with a higher dental caries prevalence and gingival problems.^9^, ^10^, ^11^

Beside the general consensus on the crucial role of folate and vitamin B12 on oral health during critical life periods such as malnutrition or high stress experiences, weak scientific evidence supports the numerous recommendations written about the importance of B vitamins supplements for dental health.^12^, ^13^ Folic acid, a synthetic dietary supplementation converted into folate by the body, plays a protective role in the occurrence of neural tube defects (NTDs).^14^, ^15^ However, high doses of folic acid supplementation in pregnancy may increase the risk of the occurrence and/or recurrence of cleft lip and/or palate, the most common congenital defects of the craniofacial structure that share similar causes with developmental abnormalities of teeth.^16^, ^17^ The consequences of high doses of folic acid supplementation in pregnancy on the child dental development are unravelled in the literature.

The conversion of folic acid into folate is catalysed by methylenetetrahydrofolate (MTHFR) enzyme which is also important in regulating folate, vitamin B12 and homocysteine values.^18^ MTHFR enzyme is encoded by the single nucleotide polymorphism *C677T* (rs1801133) of methylenetetrahydrofolate reductase gene (*MTHFR*).^19^ Accordingly, homozygous rs1801133 (TT) individuals have almost 30% of the expected MTHFR enzyme activity, implying low efficiency of processing the folic acid into folate, and a marked increase in plasma homocysteine concentration that leads to several adverse health outcomes; heterozygotes rs1801133 (CT) have almost 65% activity, and individuals with the most common genotype rs1801133 (CC) have complete MTHFR enzyme activity.^20^

Studying prenatal factors that accelerate or decelerate child dental development could facilitate the recognition of developmental abnormalities of the dentition and improve treatment planning for the right time for orthodontic intervention. In this perspective, investigation of the relationship between B vitamins and the timing of dental development is important to ascertain the role of B vitamins in dental health and to provide evidence in support of the dietary guidelines. Accordingly, we studied, in a population‐based prospective cohort study among 3728 mothers and their children, the associations of maternal folic acid supplementation and folate and vitamin B12 concentrations with child dental development. We also investigated the modifying effect of MTHFR‐C677T polymorphism on the studied associations.

## 2 MATERIALS AND METHODS

### 2.1 Study design

This investigation was embedded in the Generation R Study, a multi‐ethnic population‐based prospective cohort study from foetal life onwards, which was initiated to identify early environmental and genetic determinants of growth, development and health.^21^ All children were born between April 2002 and January 2006. Enrolment in the study was aimed at early pregnancy but was allowed until the birth of the child. The Generation R Study has been conducted in accordance with the World Medical Association Declaration of Helsinki, and all study phases have been approved by the Medical Ethical Committee of the Erasmus Medical Centre, Rotterdam, the Netherlands (MEC‐2012‐165).^22^ The current study is in compliance with STROBE checklist (Appendix S2).

### 2.2 Study sample

Among 8879 mothers prenatally included in the study, 8034 (90.5%) had available measurements on folic acid supplementation, folate, vitamin B12 or homocysteine concentrations. Of the 7943 singleton live‐born children from mothers with nutritional data available, 3728 (46.9%) had one dental panoramic radiograph (DPR) taken at the age of 10 years and used to determine their developmental stage of the permanent dentition (Figure S1).

### 2.3 Folic acid supplementation

Information on folic acid supplementation (0.4‐0.5 mg) and the initiation of supplementation was obtained by questionnaires at the enrolment of the study (median 14.6 weeks of gestation).^23^ Self‐reported folic acid use was categorized into three groups: (a) no use, defined as no use of folic acid at all; (b) preconception start, defined as the start of folic acid intake at any moment prior to conception; (c) start when pregnancy was known, defined as the start of folic acid intake from the moment that pregnancy was recognized but before the eighth week of gestation. Self‐reported folic acid use was validated in a subgroup using serum folate levels in the first trimester. Detailed information on folic acid intake is described elsewhere.^23^, ^24^ Information about folic acid supplementation was available for a subgroup of 3063 participants (82.2%).

### 2.4 Maternal folate and vitamin B12 concentrations

In early pregnancy (median gestational age 13.1 weeks; 95% range 10.5, 16.9), venous samples were drawn and stored at room temperature before being transported to the regional laboratory for processing and storage for future studies.^22^ Folate was measured in plasma samples and collected in evacuated tubes containing ethylenediaminetetraacetic (EDTA), whereas vitamin B12 was measured in serum samples. To analyse folate and vitamin B12 concentrations, the samples were transported to the Department of Clinical Chemistry at the Erasmus University Medical Centre (Rotterdam), in 2008. After thawing, the folate and vitamin B12 concentrations were determined using an immunoelectrochemoluminescence assay on the Architect System (Abbott Diagnostics BV). These methods are described in detail elsewhere.^25^, ^26^

### 2.5 *MTHFR‐C677T* carried by mothers

Maternal DNA was extracted from white blood cells in early pregnancy. Genotyping of *MTHFR*‐*C677T* used the TaqMan allelic discrimination assay (Applied Biosystems) and Abgene QPCR ROX mix (Abgene).^27^ Genotype data were extracted from an imputed genome‐wide association scan (1000G phase Iv3).^27^ The genotype frequencies of *MTHFR‐C677T* were 44.3% (CC), 34.9% (TC) and 8.0% (TT).

### 2.6 Assessment of dental development

Dental panoramic radiographs were taken at the age‐10 assessment as part of the Generation R Study protocol after child and parental informed consent was obtained. Dental panoramic radiographs of children were taken in a standardized manner by trained personnel with the use of a digital dental imaging unit (OP/OC 200D). Dental development was defined using the Demirjian method based on the available dental panoramic radiographs. According to the Demirjian method, the calculation of dental age is derived from the developmental stages of the teeth present in the lower left quadrant.^28^ The lower jaw was preferred over the upper jaw because of the higher bone compactness making it easier to assess precisely the developmental stages of teeth from radiographic images. The left side was arbitrarily chosen instead of the right side, since the left and right sides of mandible develop symmetrically in healthy individuals. One experienced examiner (B.D) determined the eight stages of development (1‐8) for each of the seven permanent teeth located in the lower left quadrant (excluding the third molar).^29^ In the event that permanent tooth in the left mandible was congenitally missing, the stage of development was assessed from the corresponding tooth on the right side. The obtained stages of development were weighted for boys and girls using the Dutch dental age standard.^30^ Finally, the summed dental maturity score was converted into dental age using the standard tables for each sex.

### 2.7 Covariates

Gestational age at blood sampling was noted when venous samples were drawn. Homocysteine concentration in early pregnancy was measured in plasma samples and further analysed in the same way as the maternal folate concentration. We obtained information on maternal age at enrolment, ethnicity, educational level and smoking during pregnancy from the questionnaires.^21^ Maternal energy intake (Kcal) during pregnancy was assessed at enrolment using a validated semi‐quantitative food frequency questionnaire.^31^ Ethnicity and educational level were defined according to the classification of Statistics Netherlands.^32^ For this study, we classified maternal ethnicity into Dutch and non‐Dutch. Children for whom both parents were born in the Netherlands were classified as Dutch. The child was of non‐Dutch origin if one or both of the parents had been born abroad. If the parents had been born in different countries, the country of birth of the mother determined the ethnicity. This approach has been previously described in detail.^22^ Maternal prepregnancy height and weight were self‐reported, and the prepregnancy body mass index (BMI) was calculated (kg/m^2^). Information on the child's sex was available from medical records and hospital registries. At the age‐10 assessment, child's height was determined in standing position to the nearest millimetre without shoes by a Harpendenstadiometer (Holtain Limited). Weight was measured using a mechanical personal scale (SECA). We calculated the child's BMI (kg/m^2^) using the weight and height measured at the age‐10 assessment. Hypodontia was ascertained from the dental panoramic radiographs, with those missing at least one tooth and with no sign of formation or calcification showing in the dental panoramic radiographs, categorized as case of hypodontia.

### 2.8 Statistical analyses

We calculated the Intra‐Class Correlation (ICC) to test the agreement between two independent examiners who assessed stages of development (1‐8) for each of the seven left mandibular teeth in a random subsample of 100 dental panoramic radiographs from the study sample. The ICC for the scored teeth ranged between 0.65 and 0.80, which is considered to be ‘substantial’ agreement according to the conventional criteria.^29^, ^33^ Central incisors were not taken into account due to the absence of variation in the stage of tooth development fitting with age of the children.

To study the association between folic acid supplementation in pregnancy and dental age of children, we built three generalized linear models. In Model 1, we adjusted for maternal‐related characteristics including maternal age and BMI at enrolment, ethnicity, education, smoking and energy intake during pregnancy. In Model 2, we additionally adjusted for child‐related characteristics including age, hypodontia, BMI and height. To control for the confounding effect of homocysteine, we added maternal homocysteine concentration as a confounder in Model 3. This analysis was performed for ‘folic acid supplementation before pregnancy was known (preconceptional)’and ‘folic acid supplementation when pregnancy was known’ in reference to ‘no folic acid supplementation’ (reference group). We applied the false discovery rate (FDR) method to correct for multiple testing.

The associations of maternal folate and vitamin B12 concentrations with dental age of children were analysed using three multivariate linear regression models. Model 1 adjusted for gestational age at blood sampling and all the other maternal‐related confounders; Model 2 additionally adjusted for child‐related confounders and Model 3 additionally adjusted for homocysteine concentration. In order to compare effect estimates, maternal folate and vitamin B12 concentrations were transformed into continuous standard values (Z‐scores) and were analysed per standard deviation (SD) ‘increase’. Furthermore, we explored the associations by categorizing folate and vitamin B12 concentrations into quartiles. Generalized linear models were built, following the same consecutive steps for the inclusion of confounders as described above. We applied the false discovery rate (FDR) method to correct for multiple testing. In addition, a test for trend was applied for the categories of folate and vitamin B12 in relation with dental age of children. Covariates were included in the regression models based on the previous literature or a change of >10% in effect estimates.^34^, ^35^

To assess whether associations of folic acid supplementation and folate and vitamin B12 concentrations with dental age differed by *MTHFR‐C677T* (rs1801133), sex or ethnicity, we analysed the interaction terms (Table S3). For the statistical significant interactions (*MTHFR‐C677T*; ethnicity), stratification analysis (Tables S4‐S6) was additionally performed. We stratified the analysis for folic acid intake of mothers (Table S2) and tested the association between folate concentration and dental age for each category of folic acid supplementation (no folic acid intake, folic acid supplementation when the pregnancy was known and periconceptional folic acid intake). We performed a nonresponse analysis (Table S1) by comparing the general characteristics between children with and without measurements of dental development, using t tests, chi‐square tests and Mann‐Whitney tests. The nonlinear associations were assessed by adding quadratic terms of exposures to the models. Markov Chain Monte Carlo method was used to prevent bias associated with missing data, and ten imputed data sets were generated, from which the pooled effect estimates are presented in this study (*β*; 95% CI).^36^ All observed differences were considered statistically significant with a *P* ≤ .05. All statistical analyses used Statistical Package for Social Sciences software version 21.0 (SPSS Inc), with the exception of the FDR correction, which used Statistical Analysis System (SAS 9.4) Software.

## 3 RESULTS

### 3.1 Participant characteristics

The general characteristics of the study sample are presented in Table 1. Among mothers included in this study, 64.4% reported folic acid supplement intake either when the pregnancy was known or preconception intake, or 17.8% reported no use of folic acid supplement during pregnancy. The median of maternal folate concentration was 17.90 (95% range: 6.9, 35.3) nmol/L and of vitamin B12 concentration was 173.00 (95% range: 87.0, 359.9) pmol/L. The mean dental age of children was 10.3 years (SD; 0.8). The mandibular canine, first premolar, second premolar and second molar had reached a median value of six developmental stages, while mandibular central incisor, second incisor and first molar have almost reached the final calcification, presenting a median value of 8 developmental stages in dental panoramic radiographs.

**TABLE 1:** Characteristics of participants included in the study (N = 3728)

	Value	Missing (N, %)
Maternal characteristics		
Gestational age at blood sampling (weeks)	13.1 (10.5, 16.9)	772 (20.7)
Maternal age (y)	30.8 (4.8)	—
Ethnicity (N, %)		—
Dutch	2130 (57.1)	
Non‐Dutch	1598 (42.9)	
Body mass index (kg/m^2^)	23.6 (19.5, 32.9)	21 (0.0)
Education (N; %)		142 (3.8)
No education	—	
Primary	266 (7.1)	
Secondary	1478 (39.6)	
Higher	1840 (49.4)	
Smoking (N, %)		318 (8.5)
Never smoked during pregnancy	2601 (69.8)	
Until pregnancy was known	299 (8.0)	
Continued smoking	510 (13.7)	
Calories intake (kcal)	2069.5 (1067.9, 3167.4)	778 (20.9)
Folic acid supplement (N, %)		665 (17.8)
No use	662 (17.8)	
Start when pregnancy was known	973 (26.1)	
Periconceptional start	1428 (38.3)	
Folate concentration (nmol/L)	17.9 (6.9, 35.3)	812 (21.8)
Total vitamin B12 concentration (pmol/L)	173.0 (87.0, 359.9)	926 (24.8)
Homocysteine concentration (μmol/L)	6.8 (4.9, 9.9)	838 (22.5)
MTHFR‐C677T		474 (12.7)
TT	300 (8.0)	
CC	1652 (44.3)	
CT	1302 (34.9)	
Child characteristics		
Gender (N, %)		—
Boys	1840 (49.4)	
Girls	1888 (50.6)	
Chronological age (y)	9.8 (0.4)	—
Ethnicity (N, %)		37 (1.0)
Dutch	2241 (60.1)	
Non‐Dutch	1450 (38.9)	
Weight (kg)	34.00 (26.4, 50.4)	—
Height (cm)	141.7 (6.8)	—
Body mass index (kg/m^2^)	17.0 (14.4, 23.2)	—
Dental age (y)	10.3 (0.8)	—
Stage of development for the central incisor	8 (8‐8)	—
Stage of development for the lateral incisor	8 (8‐8)	—
Stage of development for the canine	6 (5‐7)	—
Stage of development for the first premolar	6 (5‐7)	—
Stage of development for the second premolar	6 (5‐7)	—
Stage of development for the first molar	8 (7‐8)	—
Stage of development for the second molar	6 (4‐7)	—
Hypodontia (N, %)	198 (5.3)	—
Dental anomalies of position (N, %)	102 (2.7)	—

Note

Values are percentages for categorical variables, means (SD) for continuous variables with a normal distribution, or medians (95% range) for continuous variables with a skewed distribution.

Results from nonresponse analysis are given in Table S1. Folate and vitamin B12 concentrations in mothers of children with available measurements on dental development were higher than folate and vitamin B12 of mothers of children without available measurements of dental development.

### 3.2 The association between maternal folic acid supplementation and child dental age

As shown in Table 2, folic acid use when the pregnancy was known was associated with decelerated dental age of children (*β* = −0.18; 95% CI: −0.28, −0.08). When child‐related characteristics were considered in Model 2, the effect estimate decreased in absolute value; however, the association remained (*β* = −0.09; 95% CI: −0.17, −0.01). Adding maternal homocysteine concentration in Model 3 did not change the effect estimate of the association (*β* = −0.09; 95% CI: −0.17, −0.01). Similarly, preconception supplementation of folic acid was associated with decelerated dental age. The effect estimate attenuated from Model 1 (*β* = −0.23; 95% CI: −0.33, −0.14) to Model 3 (*β* = −0.12; 95% CI: −0.20, −0.04).

**TABLE 2:** The association between folic acid (FA) supplementation of mothers and dental age of children (N = 3063)

	Model 1		Model 2		Model 3		*P*‐value	p‐FDR
	*β*	95% CI	*β*	95% CI	*β*	95% CI		
FA^a^	−0.23	−0.33, −0.14	−0.11	−0.19, −0.03	−0.12	−0.20, −0.04	*.004*	*0.008*
FA^b^	−0.18	−0.28, −0.08	−0.09	−0.17, −0.01	−0.09	−0.17, −0.01	*.027*	*0.027*

Abbreviations: *β*, regression coefficient (represents the increase or decrease in years of dental age); CI, confidence interval.

Note Model 1: was adjusted for maternal age, BMI, ethnicity, education, smoking and Kcal intake during pregnancy.

Model 2: was additionally adjusted for age of child, hypodontia, child BMI and height.

Model 3: was additionally adjusted for maternal homocysteine concentration.

^a^ FA use before the pregnancy was known vs no FA use.

^b^ FA use when the pregnancy was known vs no FA use; significant *P*‐values are presented in italic font.

* p‐FDR = false discovery rate correction for multiple testing.

### 3.3 Associations of maternal folate and vitamin B12 concentrations with child dental age

*Analysed continuously per* SD *‘increase’ (* *Table* *3* *):* The association between maternal folate concentration in early pregnancy and decelerated dental age of children was shown only in Model 1 (*β* = −0.04; 95% CI: −0.07, −0.01). Maternal vitamin B12 concentration was not associated with dental age of children in any of the statistical models. Also, the stratification analysis for folic acid intake of mothers showed no association between maternal folate concentration and child dental age (Table S2).

**TABLE 3:** Associations of maternal folate and vitamin B12 concentrations with dental age of children (N = 3075)

	Model 1		Model 2		Model 3			
**1**.	***β***	**95% CI**	***β***	**95% CI**	***β***	**95% CI**	***P*‐value**	***p*‐FDR**
**Folate** (SDS)	−0.04	−0.07, −0.01	−0.02	−0.05, 0.02	−0.02	−0.05, 0.02	.342	0.492
**2**.	***β***	**95% CI**	***β***	**95% CI**	***β***	**95% CI**	*P*‐value*	***p*‐FDR**
**Folate** nmol/L							.203	
Q1 ref; 3.7‐11.4	—	—	—	—	—	—	—	—
Q2 (11.5‐17.9)	−0.07	−0.16, 0.02	−0.06	−0.13, 0.02	−0.06	−0.14, 0.03	.182	0.390
Q3 (18.0‐25.4)	−0.06	−0.14, 0.03	−0.03	−0.11, 0.05	−0.03	−0.11, 0.05	.497	0.568
Q4 (25.5‐45.3)	−0.10	−0.19, −0.02	−0.05	−0.13, 0.02	−0.05	−0.14, 0.03	.195	0.390
**3**.	***β***	**95% CI**	***β***	**95% CI**	***β***	**95% CI**	***P*‐value**	***p*‐FDR**
**Vitamin B12** (SDS)	0.02	−0.01, 0.05	0.02	−0.01, 0.05	0.03	−0.00, 0.06	.076	0.304
**4**.	***β***	**95% CI**	***β***	**95% CI**	***β***	**95% CI**	*P*‐value*	***p*‐FDR**
**Vitamin B12** pmol/L							.088	
Q1 ref; 44.0‐131.0	—	—	—	—	—	—	—	—
Q2 (132.0‐173.0)	0.03	−0.06, 0.11	0.03	−0.04, 0.11	0.03	−0.04, 0.11	.369	0.492
Q3 (174.0‐232.0)	0.01	−0.08, 0.10	0.01	−0.06, 0.09	0.02	−0.06, 0.09	.637	0.637
Q4 (233.0‐1476.0)	0.07	−0.02, 0.16	0.08	0.00, 0.17	0.09	0.01, 0.17	*.034*	0.272

Note

Model 1: was adjusted for gestational age at blood sampling, maternal age, BMI at intake, ethnicity, education, smoking and Kcal intake during pregnancy.

Model 2: was additionally adjusted for age of child, hypodontia, child BMI and height.

Model 3: was additionally adjusted for maternal homocysteine concentration.

Abbreviations: *β*—regression coefficient (represents the increase or decrease in years of dental age), CI, confidence interval, ref., reference; Q, quartile; significant *P*‐values are presented in italic font; *P*‐value^*^ is calculated from the test of trend performed for the categories of folate and vitamin B12.

*Analysed in quartile categories (* *Table* *3* *):* Considering all potential confounders (Model 3) and correcting for multiple testing (FDR), no association was shown between quartile categories of folate and vitamin B12 concentration in mothers and dental age of children.

### 3.4 The modifying effect of *MTHFR‐C677T* carried by mothers

*MTHFR‐C677T* interacted in the associations of maternal folate (*P* < .001) and vitamin B12 (*P* = .038) concentrations with dental age (Table S3). The stratified analysis for *MTHFR‐C677T* variants showed no association of maternal folate and vitamin B12 concentrations with dental age of children (Tables S4 and S5).

## 4 DISCUSSION

Although the importance of B vitamins on the formation of oral tissues of the newborn is underlined in many dietary recommendations for mothers, the scientific evidence that supports the value of various supplementation intake for dental health is weak.^13^ Our hypothesis on the association between maternal B vitamins and timing of dental development was based on the literature that emphasizes the contribution of folate and vitamin B12 to early cell formation and the impact of maternal vitamins in tooth formation and mineralization of the newborn.^6^, ^37^, ^38^, ^39^ Applying an epidemiological approach, we evaluated the associations of maternal folic acid supplementation and prenatal folate and vitamin B12 concentrations with child dental development. To the best of our knowledge, these associations have not been previously investigated in the general population. Findings from this large population‐based prospective cohort study suggest that maternal folic acid supplementation, either preconceptional or postconceptional, is associated with decelerated dental development by 1‐2 months lower dental age in children. Further, maternal folate and vitamin B12 concentrations in early pregnancy do not prove any effect on the velocity of child dental development. Lastly, the maternal *MTHFR‐C677T* polymorphism plays no modifying role in the studied associations of maternal folate and vitamin B12 with child dental development.

Women planning to conceive should follow the guidelines for a healthy diet, rich in folic acid. In addition, a daily folic acid supplement of 0.4‐0.5 mg from 1 to 3 months prior the conception it is recommended in order to reduce the risk of NTDs in the newborns.^14^, ^15^ Higher folic acid intake can lead to elevated blood concentrations of folate and unmetabolized folic acid.^40^ Several adverse health outcomes have been related to the accumulation of unmetabolized folic acid in plasma.^41^ In respect of the role of folic acid supplementation on the formation and growth of craniofacial structure, controversy exists in the literature due to the inconsistent findings.^16^ As in many studies, folic acid supplementation during pregnancy is recognized as beneficial in decreasing the risk of clefts; in other studies, it is associated with a higher risk of clefts, or no effect shown at all.^17^, ^42^, ^43^ Studies of the role of folic acid in dental development are scarce. We demonstrated an association between maternal pre‐ and postconceptional folic acid supplementation and decelerated dental development of children. The question of whether folic acid is implicated in the stimulation of inhibitors of tooth mineralization such as pyrophosphate might be a hypothetical explanation for our findings.^4^, ^44^ High levels of folic acid act as a folate antagonist after conversion to dihydrofolate, inhibiting the activity of MTHFR and explaining an expected modifying effect of *MTHFR* polymorphism.^45^ Polymorphism of maternal *MTHFR* variants interacted in the associations of maternal folate and vitamin B12 concentrations with dental age of children, although in the stratification analysis, a modifying effect of maternal *MTHFR* polymorphism was not shown. Low activity of MTHFR enzyme is related to lower folate and higher homocysteine levels. Folate is directly implicated in the methylation of homocysteine to methionine with vitamin B12 and methionine synthase as co‐enzymes. A study performed in rats showed that higher levels of maternal methionine lead to altered development of tooth germs in the newborns.^46^ Vitamin B12 and folate are important for the oral health and comfort of soft tissues.^10^ Although not much is known about the role of vitamin B12 and folate on the hard tissues of teeth, a protective effect against tooth decay and periodontal disease has been shown.^9^ We showed no association between maternal and vitamin B12 concentrations in early pregnancy and timing of dental development in children. Our hypothesis about the role of maternal vitamin B12 and folate in early pregnancy and development of permanent dentition in 10‐year‐old children was supported on the formation of the deciduous dentition. Around the age of 10, the permanent first molar and both incisors are almost fully developed while the canine, the first premolar, the second premolar and the second molar are at the halfway stage of development. The permanent second molar is not replaced by a deciduous tooth, and its development starts around the age of 3 years, which is quite far from the time when blood concentrations were measured or from the time when mothers reported supplementation of folic acid. If existent, the effect of vitamin B12 and folate in early pregnancy could be reflected in the timing of development of the mandibular canine, first premolar and second premolar, consequently. These permanent teeth replace the deciduous mandibular canine, first molar and second molar around the age of 10‐to‐12 years old.^47^ The formation of the deciduous canine, first molar and second molar starts around 16th‐19th week of gestation, a susceptible time for the continuation of the maturation.^47^ The initiation of formation of these teeth coincides approximately with the time when folate and vitamin B12 concentration was ascertained. Therefore, if present, the effect of folate and vitamin B12 concentrations on the development of the successors of these teeth would have been shown.

We undertook this investigation using a large prospective population‐based cohort design, which is the main strength of our study. The population‐based sample allows broader generalizability of the findings, and the prospective nature of the study leads to better control of the confounders. The information obtained for maternal folate and vitamin B12 concentrations increased the validity of the measurements due to the higher precision. However, the blood measurements were available only in one time point and they cannot be representative for the B vitamins status of mothers throughout the whole pregnancy. Longitudinal measurements of maternal B vitamins during pregnancy could assess the long‐term status of folate and vitamin B12. However, this was not possible for the current study. Detailed information in the questionnaire about the combination of folic acid supplements with or without other vitamins, on the dose of folic acid and on the duration of supplementation, was not known in our study, implying an important limitation to provide a thorough explanation of the relation of folic acid intake in pregnancy and decelerated dental development of children. Maternal folate and vitamin B12 concentrations were determined after thawing cycles, which can lead to small shifts in the values of folate and vitamin B12 concentrations, not excluding the possibility for differential misclassification bias, consequently.^48^ Although this is a unique cohort study with available large data on dental development in 10‐year‐old children, a longitudinal approach to dental development should be considered for future investigations. In this observational study, we adjusted for many potential maternal and child confounders, however, in a time span of 10 years between the measurements of the exposure and the outcome residual confounding from unmeasured factors can still be present. For example, additional nutritional factors and different lifestyle of mothers, and child nutritional status linked to breastfeeding and/or age of weaning, hormones such as parathyroid hormone (PTH) were not taken in consideration and thus should be counted as a limitation. Furthermore, the present study sample comprises relatively healthy women with a percentage of folic acid supplement use which is higher than in other populations. This might have resulted in smaller observed differences and might limit the generalizability to other populations. Also, selection bias cannot be excluded because it is difficult to assess whether the associations of folate and vitamin B12 concentrations with dental development of children were different between those included and those not included from the final analyses.

In conclusion, the findings from this observational study show that, in the general population, maternal folic acid supplementation is associated with decelerated dental development of children whereas folate and vitamin B12 concentrations in first trimester of pregnancy are not associated with the timing of child dental development. Since the delay on dental development varies between 1 and 2 months dental age, our findings do not suggest to implement changes at the existing maternal folic acid supplementation guidelines. Future research studies applying a longitudinal approach and focused at subgroups of mothers with B vitamins deficiency are needed to add scientific evidence to the current maternal dietary guidelines on the role of B vitamins on child dental development.

## CONFLICT OF INTEREST

The authors declare that there are no conflicts of interest.

## AUTHOR CONTRIBUTION

All authors have made substantial contributions to conception and design of the study. BD has been involved in data collection and data analysis. BD, VWVJ, EAPS, EBW and EMO have been involved in data interpretation, drafting the manuscript and revising it critically and have given final approval of the version to be published.

## Supporting information

Appendix S1

Appendix S2
