# Supplementary material for: The association of maternal folic acid supplementation and prenatal folate and vitamin B12 concentrations with child dental development
Source: Community Dent Oral Epidemiol. 2021 Jan 24;49(5):445–53. doi: 10.1111/cdoe.12620 (PMC8518742; doi:10.1111/cdoe.12620)
Supplement: Supplementary file 1 — Appendix S1 [file CDOE-49-445-s002.docx]

**APPENDIX S1**

**The association of maternal folic acid supplementation and prenatal folate and vitamin B12 concentrations with child dental development**

**Brunilda Dhamo ^1,2*^,Vincent WV Jaddoe ^2,3^, Eric AP Steegers ^2,4^, Eppo B Wolvius ^1,2^, Edwin M Ongkosuwito ^1^**

**^1^**Department of Oral & Maxillofacial Surgery, Special Dental Care and Orthodontics, Erasmus University Medical Centre, Rotterdam, the Netherlands ; **^2^**The Generation R Study Group, Erasmus University Medical Centre, the Netherlands; ^3^ Department of Epidemiology, Erasmus University Medical Centre, the Netherlands; ^4^ Department of Obstetrics & Gynecology, Erasmus University Medical Centre, Rotterdam, the Netherlands

***Corresponding author**: Brunilda Dhamo

Department of Oral & Maxillofacial Surgery, Special Dental Care and Orthodontics

Erasmus University Medical Centre

PO Box 2040, 3000 CA Rotterdam, the Netherlands

Tel +31 10 7036426

**Email**:b.dhamo@erasmusmc.nl

**Supplementary Figure 1.** Flowchart of study participants

Mothers with available information on folic acid supplementation and folate or vitamin B_12_ concentrations **N = 8,034**

N = 5,562

**N = 91** excluded due to twin births

Singleton life born children

**N = 7,943**

**N = 3,131** excluded children who did not attend follow up visits at the age-10 assessment

Children participating at age-10 follow up measurements

**N = 4,812**
N = 3,877

**N = 1,084** excluded children without DPR available or bad image

Final population for analysis

with available measurements on folic acid supplementation and folate or vitamin B12 concentrations and dental development of children **N=3,728**

| **Supplementary Table 1.** Characteristics of non-participants in the follow-up measurements of dental development included in the study (N=3728) | | | |
| --- | --- | --- | --- |
| ***Maternal characteristics*** | **Participation**  (N=3728) | **No-participation** (N=1084) | **p-value** |
| Gestational age at blood sampling (weeks) | 13.1 (10.5, 16.9) | 13.5 (9.7, 35.6) | *<0.001* |
| Missing (N, %) | 772 (20.7) | 272 (25.1) |  |
| Maternal age (years) | 30.8 (4.8) | 30.6 (5.1) | 0.309 |
| Ethnicity (N, %) |  |  | 0.863 |
| Dutch | 2130 (57.1) | 619 (57.1) |  |
| Non-Dutch | 1598 (42.9) | 448 (41.3) |  |
| Missing (N, %) | - | 17 (1.6) |  |
| Body mass index (kg/m^2^) | 23.6 (19.5, 32.9) | 23.9 (18.7, 35.6) | 0.187 |
| Missing (N, %) | 21 (0.0) | 4 (0.0) |  |
| Education (N; %) |  |  | *0.040* |
| No education | - | 3 (0.00) |  |
| Primary | 266 (7.1) | 76 (7.0) |  |
| Secondary | 1478 (39.6) | 472 (43.5) |  |
| Higher | 1840 (49.4) | 487 (44.9) |  |
| Missing | 142 (3.8) | 184 (17.0) |  |
| Smoking (N, %) |  |  | 0.576 |
| Never smoked during pregnancy | 2601 (69.8) | 758 (69.9) |  |
| Until pregnancy was known | 299 (8.0) | 96 (8.9) |  |
| Continued smoking | 510 (13.7) | 161 (14.9) |  |
| Missing | 318 (8.5) | 69 (6.4) |  |
| Calories intake (kcal) | 2069.5 (1067.9, 3167.4) | 1976.3 (952.5, 3218.3) | *0.002* |
| Missing (N, %) | 778 (20.9) | 167 (15.4) |  |
| Folic acid supplement (N, %) |  |  | *0.037* |
| No use | 662 (17.8) | 230 (21.2) |  |
| Start when pregnancy was known | 973 (26.1) | 291 (26.8) |  |
| Preconception start | 1428 (38.3) | 379 (35.0) |  |
| Missing | 665 (17.8) | 184 (17.0) |  |
| Folate concentration (nmol/l) | 17.9 (6.9, 35.3) | 14.8 (5.7, 36.6) | *<0.001* |
| Missing (N, %) | 812 (21.8) | 285 (26.3) |  |
| Vitamin B12 concentration (pmol/l) | 173.0 (87.0, 359.9) | 171.0 (73.9, 387.3) | *<0.001* |
| Missing (N, %) | 926 (24.8) | 330 (30.4) |  |
| Homocysteine concentration (μmol/l) | 6.8 (4.9, 9.9) | 7.2 (4.6, 13.4) | *<0.001* |
| Missing (N, %) | 838 (22.5) | 296 (27.3) |  |
| *MTHFR-C677T* |  |  | 0.778 |
| TT | 300 (8.0) | 74 (6.8) |  |
| CC | 1652 (44.3) | 435 (40.1) |  |
| CT | 1302 (34.9) | 354 (32.7) |  |
| Missing | 474 (12.7) | 221 (20.4) |  |
| ***Child characteristics*** | **Participation**  (N=3728) | **No-participation** (N=1084) | **p-value** |
| Gender (N, %) |  |  | 0.367 |
| Boys | 1840 (49.4) | 528 (48.7) |  |
| Girls | 1888 (50.6) | 556 (51.3) |  |
| Chronological age (years) | 9.8 (0.4) | 9.8 (0.5) | *0.006* |
| Ethnicity (N, %) |  |  | 0.324 |
| Dutch | 2241 (60.1) | 643 (59.3) |  |
| Non-Dutch | 1450 (38.9) | 425 (39.2) |  |
| Missing (N, %) | 37 (1.0) | 16 (1.5) |  |
| Weight (kg) | 34.0 (26.4, 50.4) | 33.6 (25.0, 53.4) | 0.196 |
|  | - | 132 (12.2) |  |
| Height (cm) | 141.7 (6.8) | 141.2 (6.5) | 0.066 |
|  | - | 133 (12.3) |  |
| Body mass index (kg/m2) | 17.0 (14.4, 23.2) | 17.0 (13.9, 25.2) | 0.457 |
|  | - | 133 (12.3) |  |

| Values are percentages for categorical variables, means (SD) for continuous variables with a normal distribution, or medians (95% range) for continuous variables with a skewed distribution; Differences were tested using independent t-test for continuous variables, chi-squared test for categorical variables and Mann-Whitney Non-Parametric test for variables with a skewed distribution, using participation group as the reference; Significant p-values are presented in italic font |
| --- |

| **Supplementary Table 2.** The association between maternal folate concentration and dental age of children stratified for maternal FA intake | | | | | | |
| --- | --- | --- | --- | --- | --- | --- |
|  | **Model 1** | | **Model 2** | | **Model 3** | |
| **No FA intake** | **β** | **95% CI** | **β** | **95% CI** | **β** | **95% CI** |
| Folate (SDS) | -0.06 | -0.16, 0.04 | -0.04 | -0.13, 0.05 | -0.05 | -0.15, 0.05 |
| **FA ^a^** | **β** | **95% CI** | **β** | **95% CI** | **β** | **95% CI** |
| Folate (SDS) | -0.03 | -0.11, 0.05 | -0.01 | -0.06, 0.04 | -0.01 | -0.06, 0.04 |
| **FA ^b^** | **β** | **95% CI** | **β** | **95% CI** | **β** | **95% CI** |
| Folate (SDS) | 0.02 | -0.04, 0.07 | 0.03 | -0.03, 0.08 | 0.02 | -0.03, 0.08 |
| Abbreviations: FA –folic acid, β –regression coefficients, CI – confidence interval; ^a^ Folic acid use before pregnancy was known (preconception); ^b^ Folic acid use when pregnancy was known;  Model 1: was adjusted for gestational age at blood sampling, maternal age, BMI at intake, ethnicity, education, smoking and Kcal intake during pregnancy  Model 2: was additionally adjusted for age of child, hypodontia, child BMI and height  Model 3: was additionally adjusted for maternal homocysteine concentration | | | | | | |

| **Supplementary Table 3**. Interactions of sex, ethnicity and maternal MTHFR-C677T with maternal FA supplementation and maternal folate and vitamin B12 concentrations in relation to dental age | | |
| --- | --- | --- |
| maternal *MTHFR-C677T* |  | p-value |
|  | FA supplementation | 0.146 |
|  | Folate concentration (nmol/l) | *<0.001* |
|  | Total vitamin B12 concentration (pmol/l) | *0.038* |
| Sex |  |  |
|  | FA supplementation | 0.125 |
|  | Folate concentration (nmol/l) | 0.070 |
|  | Total vitamin B12 concentration (pmol/l) | 0.725 |
| Ethnicity |  |  |
|  | FA supplementation | 0.137 |
|  | Folate concentration (nmol/l) | *0.001* |
|  | Total vitamin B12 concentration (pmol/l) | 0.609 |
| One linear regression model was built containing the independent variable (FA supplementation, folate, or vitamin B12 concentrations), the covariate (maternal *MTHFR-C677T* variant, sex or ethnicity) and the interaction term between the two. The significant p-values for each interaction term are presented in italic font. | | |

| **Supplementary Table 4**. The association between maternal folate concentration and dental age of children stratified for maternal *MTHFR-C677T* variants | | | | | | |
| --- | --- | --- | --- | --- | --- | --- |
|  | **Model 1** | | **Model 2** | | **Model 3** | |
| **1. CC** **variant (N=1337)** | **β** | **95% CI** | **β** | **95% CI** | **β** | **95% CI** |
| Folate (SDS) | -0.05 | -0.10, 0.00 | -0.03 | -0.07, 0.02 | -0.02 | -0.07, 0.03 |
| **2.** **TT or CT variant (N=1329)** | **β** | **95% CI** | **β** | **95% CI** | **β** | **95% CI** |
| Folate (SDS) | -0.04 | -0.08, 0.01 | -0.01 | -0.05, 0.03 | -0.01 | -0.05, 0.03 |
| *Abbreviations:* β –regression coefficients, CI – confidence interval, Q-quartile; significant p-values are presented in italic font  Model 1: was adjusted for gestational age at blood sampling, maternal age, BMI at intake, ethnicity, education, smoking and Kcal intake during pregnancy  Model 2: was additionally adjusted for age of child, hypodontia, child BMI and height  Model 3: was additionally for maternal homocysteine concentration  *Addition:* The medians of folate concentration did not differ across groups of mothers who carried the least frequent TT variant (median, 95% range;17.95, 6.0-33.7 nmol/L), CC variant (median, 95% range; 17.50, 6.3-37.4 nmol/L) and CT variant (median, 95% range; 18.40, 6.3-39.5 nmol/L) | | | | | | |

| **Supplementary Table 5.** The association between maternal vitamin B12 concentration and dental age of children stratified for maternal *MTHFR-C677T* variants | | | | | | |
| --- | --- | --- | --- | --- | --- | --- |
|  | **Model 1** | | **Model 2** | | **Model 3** | |
| **1.** **CC variant (N=1337)** | **β** | **95% CI** | **β** | **95% CI** | **β** | **95% CI** |
| Vitamin B12 (SDS) | 0.03 | -0.01, 0.07 | 0.03 | -0.01, 0.07 | 0.03 | -0.01, 0.07 |
| **2.** **CT or TT variants (N=1329)** | **β** | **95% CI** | **β** | **95% CI** | **β** | **95% CI** |
| Vitamin B12 (SDS) | 0.00 | -0.04, 0.05 | 0.02 | -0.03, 0.06 | 0.02 | -0.02, 0.06 |
| *Abbreviations:* β –regression coefficients, CI – confidence interval, ref.-reference; Q-quartile; significant p-values are presented in italic font  Model 1: was adjusted for gestational age at blood sampling, maternal age, BMI at intake, ethnicity, education, smoking and Kcal intake during pregnancy  Model 2: was additionally adjusted for age of child, hypodontia, child BMI and height  Model 3: was additionally adjusted for maternal homocysteine concentration  *Addition:* The medians of vitamin B12 concentration did not differ across groups of mothers who carried the least common TT variant (median, 95% range; 175.00, 70.8-343.0 pmol/L), CC variant (median, 95% range; 170.00, 76.0-418.4 pmol/L) and CT variant (median, 95% range; 174.00, 76.1-434.5 pmol/L). | | | | | | |

| **Supplementary Table 6**. The association between maternal folate and dental age stratified for ethnicity | | | | | | |
| --- | --- | --- | --- | --- | --- | --- |
|  | **Model 1** | | **Model 2** | | **Model 3** | |
| **1.Dutch** | **β** | **95% CI** | **β** | **95% CI** | **β** | **95% CI** |
| Folate (SDS) | -0.02 | -0.06, 0.02 | -0.01 | -0.05, 0.03 | -0.01 | -0.05, 0.03 |
| **2.non-Dutch** | **β** | **95% CI** | **β** | **95% CI** | **β** | **95% CI** |
| Folate (SDS) | -0.06 | -0.11, -0.01 | -0.03 | -0.07, 0.02 | -0.02 | -0.08, 0.03 |
| *Abbreviations*: β –regression coefficients, CI – confidence interval, Q-quartile; significant p-values are presented in italic font  Model 1: was adjusted for gestational age at blood sampling, maternal age, BMI at intake, ethnicity, education, smoking and Kcal intake during pregnancy  Model 2: was additionally adjusted for age of child, hypodontia, child BMI and height  Model 3: was additionally adjusted for maternal homocysteine concentration  *Addition*: Folate concentration in non-Dutch mothers (median, 95% range;14.10, 5.7-37.8 nmol/L) was lower (p<0.001) than in Dutch mothers (median, 95% range; 19.90, 6.8-39.1 nmol/L). | | | | | | |
